# Special Issue “Personalized Medicine in Blood Disease of Children”

**DOI:** 10.3390/jpm14030285

**Published:** 2024-03-05

**Authors:** Adriana Ceci, Petros Kountouris, Antonella Didio, Fedele Bonifazi

**Affiliations:** 1 Fondazione per la Ricerca Farmacologica Gianni Benzi Onlus, 70010 Valenzano, Italy; ad@benzifoundation.org (A.D.); fb@benzifoundation.org (F.B.); 2 Molecular Genetics Thalassaemia Department, The Cyprus Institute of Neurology & Genetics, Nicosia 2371, Cyprus; petrosk@cing.ac.cy

## 1. Special Issue Overview

Personalized medicine is defined as a medical model using the characterization of individuals’ phenotypes and genotypes (e.g., molecular profiling, medical imaging, and lifestyle data) for tailoring the right therapeutic strategy for the right person at the right time and/or to determine the predisposition to disease and/or to deliver timely and targeted prevention [1].

This Special Issue entitled “Personalized Medicine in Blood Disease of Children” aims to highlight the current state of the research and practice on personalized medicines in child non-malignant blood disorders. This is a research field encompassing significant advances over the past decade, including the emergence of gene therapy and other innovative therapies [2].

This Special Issue includes a collection of seven articles, focused on, in particular, hemoglobinopathies, spanning different countries worldwide such as Australia, Cyprus, Germany, Indonesia, Italy, Kuwait, Malaysia, the Philippines, South Africa, and the United Kingdom. Three out of the seven articles included in this Special Issue address topics related to sickle cell disease (SCD), while one article focusses on thalassemia only. Three articles handle both thalassemia and SCD. Three articles cover non-clinical aspects, i.e., the genetic and molecular basis of hematological diseases, particularly the use of omics and the identification of genetic variants possibly leading to successful diagnostic and therapeutic drug candidates. Clinical aspects are explored in four articles, ranging from screening to diagnostic and therapeutic approaches.

## 2. Genetic and Molecular Basis of Hematological Diseases

Adekile A. et al. provided insights on the role of next-generation sequencing (NGS) in hemoglobinopathies, focusing on 159 patients with sickle cell anemia and 68 Sβ-thalassemia patients, previously diagnosed through the use of high-performance liquid chromatography. The authors stressed that for complete and comprehensive disease management, it is necessary to determine each patient’s genotype, considering the different polymorphisms that significantly modulate the phenotype and predispose the individual to, or protect them from, different complications [3].

The role of genotyping in the improvement of the screening, care, treatment, and prevention of blood diseases was further explored by Akbulut-Jeradi N. et al., who reported four previously unpublished variants showing significant association with hemoglobin F (HbF). HbF is one important modifier of the SCD phenotype that impedes the polymerization of sickle hemoglobin (HbS), leading to a significant improvement in the clinical course of the disease. The authors investigated the association between single nucleotide polymorphisms and HbF levels through the use of NGS in 237 Kuwaiti SCD patients, divided into three subgroups according to their HbF levels [4].

These articles underline the fact that the phenotype has a multi-genic basis and NGS can be deployed to simultaneously screen relevant panels to afford personalized, evidence-based counseling and early intervention [3,4].

## 3. Predictive Value of Patients’ Genotype

In addition, Meloni A. et al. found an association between genotype and cardiac and pancreatic iron overload in pediatric patients with transfusion-dependent β-thalassemia enrolled in the Extension-Myocardial Iron Overload in Thalassemia Network. Iron overload represents one of the main concerns of long-term blood transfusion therapy, required for patients with thalassemia syndromes. The authors underlined that knowledge of patients’ genotypes can be valuable in predicting phenotypic features and guiding the clinical management of these patients [5].

## 4. Age-Related SCD Specificities

SCD in pregnancy remains associated with both maternal morbidity and fetal/neonatal morbidity and mortality. This was confirmed by a monocentric study conducted by Proske P. et al. on 46 pregnancies in Germany. The authors identified a high rate of maternal complications in pregnant SCD patients including vaso-occlusive crises/acute pain crises, acute chest syndrome, transfusion demand, urinary tract infections, and thromboembolic events. Neonatal growth was additionally explored [6].

In the management of these hematological chronic diseases, transition care aims to provide continuous high-quality medical care without interruption. Alashkar F. et al. provided the first multicenter German consensus statement, developed by an expert panel and addressing the importance of implementing a standardized transition guideline that allows adolescents and young adults with SCD to safely transition from pediatric to adult care in Germany [7].

## 5. Screening Programs Worldwide

Preventive screening programs are also crucial to identifying carriers of hemoglobin disorders in the population to assess the risk of having children with a severe form of the disease and to reduce the prevalence of hemoglobinopathies.

Screening programs have been implemented in several countries worldwide. Rouh AlDeen N. et al. presented the results of a study aimed at estimating the prevalence rates of β-thalassemia and SCD and disease carriers in a large adult population screened as part of the Kuwaiti National Premarital Screening Program. Notwithstanding, the program succeeded in identifying high-risk couples, yet more efforts are needed to improve the program and intensify the counseling provided, strengthen the awareness of the general population, and induce earlier age screening policies [8].

## 6. Interdisciplinarity to Cover Disease Complexity

Furthermore, Halim-Fikri B.H. et al. highlight the crucial role of interdisciplinary and international collaboration to improve the management of hemoglobinopathies and the overall disease burden. Within the Global Globin Network, the authors proposed a universally applicable system for evaluating and clustering countries, based on qualitative indicators according to the quality of care, treatment, and prevention of hemoglobinopathies. Four groups of countries were identified based on data accessible on the ITHANET portal [9]. This work will help analyze and monitor the services and epidemiology for hemoglobinopathies globally and will make recommendations for experts on database development and disease management [10].

## 7. Summary

The main findings of this Special Issue can be summarized as follows.

SCD is the most represented hemoglobinopathy in this Special Issue. This is in line with the emerging relevance of SCD in that it is the most frequent monogenic inherited disease affecting millions of people around the world. The papers collected as a part of this Special Issue, document, in particular, the increasing awareness of the disease’s seriousness and complexity in those regions where health strategies and prevention and treatment policies are well developed as well.

Together, these papers underline the fact that genetic research is crucial to enhancing both diagnostic capacity and clinical management. In fact, most papers draw particular attention to the genetic basis and advancements of genotyping in the interpretation of phenotypic variations with variable clinical outcomes. Three out of the seven papers aim to determine the impact of genetic data and clinical features also supporting disease management. This is also reflected by the role of genomics in personalized medicine for hemoglobinopathies in several ongoing international initiatives, such as the NHLBI TOPMed Program [11], the International Hemoglobinopathy Research Network (INHERENT) [12], and GenoMed4All [13].

Moreover, it appears from specific papers included in this Special Issue, that, in the case of SCD, the prevention approach, by means of prenatal or postnatal screening, is a problem not already solved and still conflicting, with ethical, religious, political, and social issues in some countries.

However, other topics may be further implemented in the future, i.e., clinical studies in special populations such as children, the development of appropriate policies including the availability of funding for innovative technologies, accelerated access to advanced therapy medicinal products, and patients centrality in research and in medical care organization.
